# Cholemic Nephrosis: An Autopsy Study of a Forgotten Entity

**DOI:** 10.5146/tjpath.2021.01532

**Published:** 2021-09-15

**Authors:** Valli Priyaa, Bheemanathi Hanuman Srinivas, Debasis Gochhait, Rajesh Nachiappa Ganesh, Bhawana A Badhe, P S Priyamvada, Deepak Amalnath, Siddhartha Das, Kusa Kumar Shaha

**Affiliations:** Department of Pathology, Indira Gandhi Medical College and Research Institute, Puducherry, India; Department of Pathology, Jawaharlal Institute of Postgraduate Medical Education and Research, Puducherry, India; Department of Nephrology, Jawaharlal Institute of Postgraduate Medical Education and Research, Puducherry, India; Department of Medicine, Jawaharlal Institute of Postgraduate Medical Education and Research, Puducherry, India; Department of Forensic Medicine and Toxicology, Jawaharlal Institute of Postgraduate Medical Education and Research, Puducherry, India

**Keywords:** Bile, Toxin, Nephropathy

## Abstract

***Objective:*** The aim of the study is to do a clinicopathologic study of post mortem kidney biopsies with significant deposition of bilirubin pigment within tubular epithelial cells and in the lumen of distal tubules as a bile cast.

***Material and Method:*** All post mortem specimens with acute tubular necrosis, with the presence of bile casts in tubules or bile pigment deposition in the tubular epithelium during the period 2015-2018 were examined for gross and histopathology along with biochemical parameters and viral markers.

***Results:*** Bile casts with sloughed renal tubular epithelial cells and occasional macrophages were present in the distal convoluted tubule in 78.6% of biopsies (11/14). The plugging of distal convoluted tubule with casts was similar to that seen in myeloma and myoglobin cast nephropathies. Bilirubin pigment deposition was present in 35.7% (5/14) of cases. The frequency of bile casts in each biopsy was variable and it did not have any association with serum bilirubin levels or etiology of liver dysfunction. A striking difference from earlier studies is the high number of toxin-induced liver damage including six cases of paraquat and 2 cases of yellow phosphorus poisoning.

***Conclusion:*** This study proves importance of the bile cast nephropathy as a reason for kidney injury, especially with varied hepatotoxic etiologies, especially paraquat and yellow phosphorus.

## INTRODUCTION

Acute kidney injury as a result of hepatic failure is a well-known entity. Hepatorenal syndrome (HRS) (Type I and Type II), a functional, potentially reversible form of renal failure resulting from severe vasoconstriction of the splanchnic vascular bed, is considered as the central pathophysiologic mechanism behind the kidney failure (1,2). However, it is reported that only 50% of patients respond to the standard treatment protocol for HRS. Hence factors other than a functional disorder might be operational (3–5).

Cholemic (bile) nephrosis or bile cast nephropathy is an overlooked etiology for kidney injury in liver failure. Even though this entity was described as early as the 1940s, subsequently, the term had almost disappeared from medical literature (6). The last decade witnessed a resurgence in information, mostly as case reports and case series (7,8). Cholemic (bile) nephrosis refers to renal dysfunction as a result of injury to renal tubules due to bilirubin, along with a spectrum of morphological changes. Cholephils other than bilirubin, including bile acids, are believed to be nephrotoxic (9,10). Mouse experiments with bile duct ligation have proved the same (11). Even though the traditional descriptions came from post mortem studies in subjects with alcoholic cirrhosis, bile casts have been described in other conditions like biliary cirrhosis and acute on chronic liver failure (ACLF) (12,13). These casts cause tubular obstruction and toxicity in a manner akin to that of myeloma or myoglobin casts (1). Bilirubin levels were variably correlated to the presence of bile casts. A firm counter-argument is that these casts might be an incidental finding resulting from reduced glomerular filtration and urinary stasis (14). The current understanding of cholemic nephrosis is incomplete due to the lack of kidney biopsy studies in hepatic failure. In this series, we describe the histopathology of cholemic nephrosis and its correlation with the etiological and biochemical parameters.

The aim of this study is to do a clinicopathologic study of post mortem kidney biopsies with significant deposition of bilirubin pigment within tubular epithelial cells and in the lumen of distal tubules as a bile cast.

## MATERIALS and METHODS

Data was taken from the medical records maintained by the department of pathology. All post mortem specimens with acute tubular necrosis, with the presence of bile casts in tubules or bile pigment deposition in the tubular epithelium during the period 2015-2018, were included. The data was collected in a predefined proforma. The post mortem examinations were conducted either as a medicolegal requirement or because of a pathological autopsy requested by the treating clinician. The specimens were retrieved and analyzed by two pathologists blind to the clinical data. Gross examination of the liver and both kidneys was done, followed by microscopic examination. Two sections each were studied for liver and kidneys, and they were stained with H&E, PAS, Perls, and Hall’s stain. Reticulin and Masson’s trichrome stains were also performed on liver biopsy to assess the architecture and fibrosis. Liver architecture, type of hepatocyte injury, portal tract changes, and presence/absence of cirrhosis were studied. Kidney biopsies were assessed for presence of bile pigment/casts, glomerular changes, acute tubular injury, interstitial scarring, and blood vessel changes. The antemortem clinical details, including the etiology of liver failure and duration, liver and renal function tests, viral markers, and urine examination results were collected wherever available.

## RESULTS

A total of 14 cases of bile cast nephropathy/bile nephrosis were identified during the study period. The median age was 30(IQR: 23-52). The etiology for hepatic failure was alcoholic cirrhosis (3), biliary cirrhosis (1), toxins (8), snake envenoming (1), and fulminant hepatitis (1). Antemortem investigations were available for twelve patients.

### Biochemical Parameters

All the patients had hyperbilirubinemia with elevated liver enzymes. All patients tested negative for Hepatitis A, B, C, E, and Epstein Barr virus. Serum creatinine median was 2.9(IQR, 1.1-2.4). Proteinuria and hematuria were noted in three and two patients, respectively. The proteinuria was subnephrotic, and quantification was not done. Microscopy of urine sediment showed the presence of bile cast in one case, and granular casts in two cases. Pyuria was present in five of seven cases (Table 1).

**Table 1:** Biochemical investigations in the fourteen cases of bile cast nephropathy

Liver and renal function tests (n = 12*)	**Median (IQR)**
**S. Bilirubin (mg)**	18.6 (10.2-23.4)
**AST(IU)**	193 (110-323)
**ALT (IU)**	183 (71 – 276)
**ALP (IU)**	758 (341-999)
**S. Creatinine (mg/dl)**	2.9

*Biochemical parameters were available for 12/14 patients **IQR:** Interquartile range.

### Gross Examination and Histopathology

Micronodular cirrhosis was present in 6 cases, while the remaining cases showed only greenish color with bile staining. No focal lesions were identified in the liver. The kidneys also showed bile staining (yellowish-green) in 8 cases predominantly in the medulla in contrast to the cortex. The rest was unremarkable on gross examination. None of them showed evidence to suggest glomerulonephritis.

**Figure 1:**
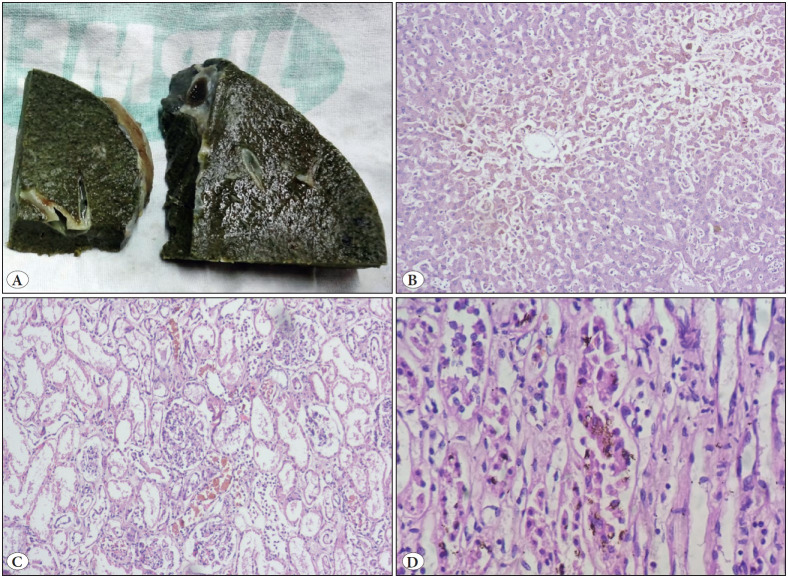
Case 3: A case of paraquat poisoning demonstrating **A)** Greenish discoloration of liver. **B)** Liver biopsy demonstrating cholestasis and central venous congestion (H&E; x100). **C)** Kidney biopsy demonstrating bile pigment within proximal tubule (H&E; x200). **D)** Kidney biopsy with bile casts in distal tubules (H&E; x400).

**Figure 2:**
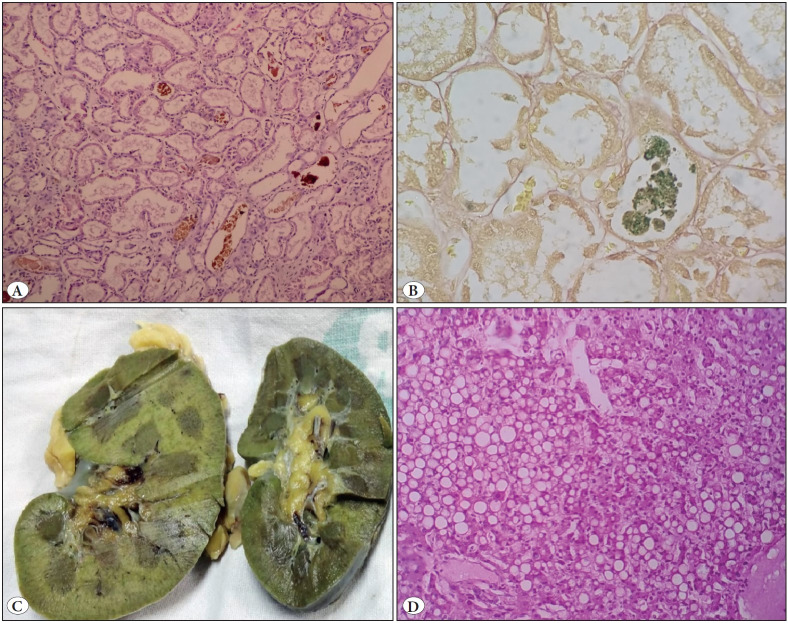
Case 5: A case of paraquat poisoning: **A)** Bile cast within distal tubules (H&E; x200). **B)** Bile cast highlighted emerald green in Hall stain (Hall’s stain; x400). **C)** Greenish discolouration of kidney due to oxidation of bilirubin to biliverdin. **D)** Liver biopsy showing macrovesicular steatosis (H&E; x200).

**Figure 3:**
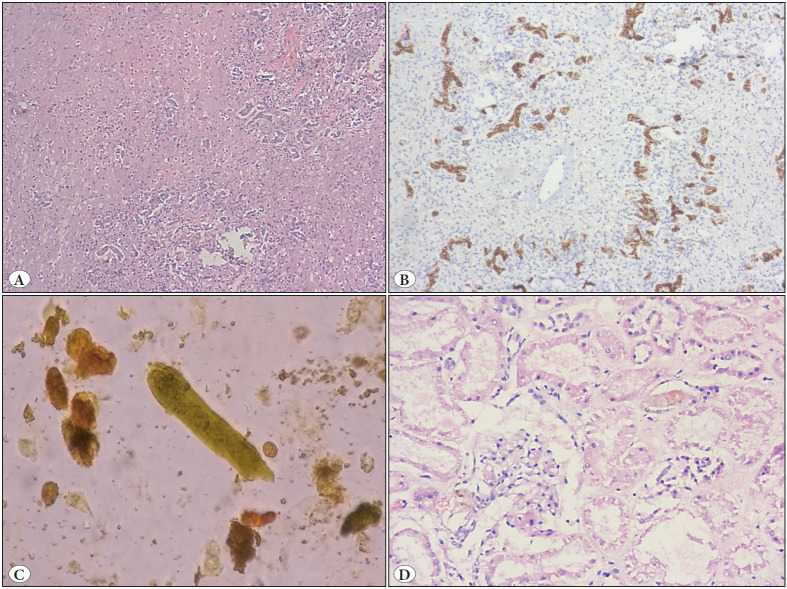
Case 14: Yellow phosphorus poisoning with **A)** Liver biopsy demonstrating bile ductular proliferation (H&E; x100). **B)** Liver biopsy with bile ductular proliferation (CK19 immunostain; x200). **C)** Urine sediment examination showing bile cast along with sloughed epithelial cells(Wet mount; x400). **D)** Kidney biopsy demonstrating acute tubular necrosis with loss of brush border in proximal tubules (H&E; x400).

On microscopic examination, evidence of cirrhosis was present in 6 cases (alcoholic cirrhosis in 4, biliary cirrhosis and cryptogenic in one). One of these six cases also had macrovesicular steatosis, and others had mild to moderate lymphocytic infiltration in the portal tract. Six specimens with a history of rodenticide poisoning showed cholestasis along with central venous congestion (Figure 1 Figure 2). Two cases (fulminant hepatitis, snake envenoming) showed submassive necrosis. Two cases of yellow phosphorus were also studied and showed nodule formation and cholestasis in one and feathery degeneration with macrovesicular stetosis in the other (Figure 3).

The kidneys showed acute tubular injury in all 14 cases, ranging from loss of brush border to tubular epithelial cell necrosis. Only bile casts were noted in nine, bile pigments alone in three, and both bile casts and bile pigments in two. These were reddish-brown granular to greenish-yellow acellular in nature and located predominantly in the distal tubules. Hall’s stain highlighted these casts and helped differentiate from myoglobin and hemoglobin casts, which may have similar morphology. Additionally, Perl’s stain was also negative. Some of these casts were associated with sloughing of the tubular epithelium. However, giant cell reaction, as seen conspicuously in myeloma cast nephropathy, was absent. Besides, two cases revealed sharp ends on these bile casts, with PAS stain. Deposition of bile pigment along the proximal convoluted tubule was observed in five instances. Glomeruli were unremarkable in all. Age-related glomerulosclerosis and blood vessel changes were noted in five cases. Interstitial scarring in the form of tubular atrophy and fibrosis was present in four cases. The individual patient characteristics are given in Table 2.

**Table 2:** Cases of cholemic nephrosis (bile cast nephropathy) with histopathological, biochemical findings.

**No**	**Age**	**Sex**	**Etiology**	**Liver disease**	**Liver – Gross features**	**Liver histology**	**Bile cast**	**ATN**	**Bile pigment**
1	75	M	Alcoholic cirrhosis	Alcoholic cirrhosis	Greenish, nodular	Cirrhosis, Portal tract infiltrate	Absent	Present	Present
2	23	F	Paraquat poisoning	Sub massive necrosis	Greenish	Sub massive necrosis, Cholestasis	Present	Present	Absent
3	52	F	Paraquat poisoning	Cholestasis	Greenish	CVC, Cholestasis	Absent	Present	Present
4	36	M	Paraquat poisoning	Cholestasis	Greenish	CVC, Cholestasis	Present	Present	Absent
5	27	M	Paraquat poisoning	Cholestasis	Greenish	Feathery degeneration, Periportal lymphocytes, Macrovesicular steatosis	Present	Present	Present
6	9	M	Hepatitis #	Sub massive necrosis	Unremarkable	PBC	Present	Present	Absent
7	17	F	Paraquat poisoning	Cryptogenic cirrhosis	Greenish, nodular	Nodule formation	Present	Present	Present
8	58	M	Alcoholic cirrhosis	Cirrhosis	Greenish nodular	Nodule formation	Present	Present	Absent
9	63	M	Alcoholic cirrhosis with HRS	Cirrhosis	Micronodular	Nodule formation	Present	Present	Absent
10	45	M	Snake envenoming	Sub massive necrosis, early cirrhosis	Nodular	Submassive necrosis	Present	Present	Absent
11	19	F	Biliary cirrhosis	Cirrhosis, biliary,	Greenish nodular	Portal tract, ballooning degeneration	Present	Present	Absent
12	27	M	Paraquat poisoning	Cholestasis	Greenish	Cholestasis	Present	Present	Absent
13	33	F	Yellow phosphorus poisoning	Cirrhosis	Greenish	Nodule formation, steatosis, cholestasis	Present	Present	Absent
14	18	F	Yellow phosphorus poisoning	Cholestasis, Steatosis	Greenish	Feathery degeneration, Ductular proliferation, Macro vesicular steatosis	Absent	Present	Present

**ATN:** Acute tubular necrosis, **CVC:** Central venous congestion, # - Viral markers including Hepatitis A, HbSAg, HCV, CMV, EBV

## DISCUSSION

The current study examined the renal and liver pathologies in patients who succumbed to chronic liver disease and hepatic failure. The study showed the presence of bile casts and bile pigments in cirrhosis of various etiologies, cholestasis as well as hepatic necrosis. Renal pathologies, other than the hepatorenal syndrome, are often overlooked in patients with liver dysfunction (13). Performing a kidney biopsy might not be feasible in these patients considering the presence of coagulopathy. Post mortem biopsy data points towards the existence of other pathologies that would coexist in these patients. The current understanding of the role of bile as a nephrotoxin has significant lacunae owing to the limited published literature.

The resurgence of literature on the potential nephrotoxicity of bile casts started with van Slambrouck et al. reporting the presence of bile casts in around 54% of kidney biopsy specimens (1). The samples were mostly post mortem, and all subjects with alcoholic liver disease and kidney failure had bile casts in the kidney. The prevalence of bile casts was lesser among subjects with other etiologies. They also noticed that a higher proportion of subjects with a diagnosis of HRS had bile casts compared to those without HRS. They also reported considerably higher bilirubin levels in those with bile casts; however, there were no differences in the severity of renal failure or transaminitis (1).

Another post mortem series reported a prevalence of 55% in cirrhotic patients. Unlike the reports of van Slambrouck et al., they found a higher incidence of bile casts among patients with chronic liver disease secondary to HCV infection. The absolute values of bilirubin were much lower (Mean value:10 mg/dL) compared to those reported by van Slambrouck et al. (1,15). Another series by Nayak et al. reported a prevalence of 44.8%, with 2.5-fold higher frequency in subjects with acute on chronic liver failure compared to cirrhosis (13).

Again bilirubin and severity of liver failure assessed by MELS score were the only predictors of the presence of bile casts. From the above studies, it appears that the most important determinant for the presence of bile casts is the degree of hepatic dysfunction. However, it should be recalled that serum creatinine is often unreliable in subjects with liver failure, due to the lower muscle mass as well as reduced hepatic production. None of the studies have reported any correlation with urinary findings. One of the cases in the present study showed bile-stained casts along with renal tubular epithelial cells. Krones et al. also noted similar findings, and they correlated with renal biopsy findings of bile cast nephropathy (12). There are multiple case reports describing the presence of bile casts in obstructive jaundice, anabolic steroid use, etc. (12).

### Renal Gross and Biopsy Findings

Most of the studies report the greenish staining of renal parenchyma with accentuation in the medulla, which is due to the higher concentration of bilirubin in distal convoluted tubule (DCT). van Slambrouk et al. noted it in 17 % of cases and it did not correlate with any particular etiology of liver failure (1). This finding is seen in 57.1% (8/14) of our cases.

Renal biopsy showed presence of bile casts with sloughed renal tubular epithelial cells and occasional macrophages in the distal convoluted tubule in 78.6% of the biopsies (11/14). The plugging of DCT with casts was similar to that seen in myeloma and myoglobin cast nephropathies. The frequency of bile casts in each biopsy was variable and it did not have any association with serum bilirubin levels or etiology of liver dysfunction. van Slambrouk et al. noted bile casts in 51.2% (21/41) of which 15/21 were confined to the distal nephrons whereas 6 cases involved both proximal and distal nephron segments. None of the fourteen cases in the present study showed bile casts in the proximal convoluted tubule.

Nayak et al. studied cases of decompensated cirrhosis, acute on chronic liver failure (ACLF), in which 57/127 (44.8%) demonstrated bile casts (13). The other changes noted were interstitial edema (19.3%), fibrosis (10.5), and tubular atrophy (1.7%) Additionally, bilirubin deposition was seen in 35.7% (5/14) of the cases.

Initial suspicion of bile casts was made on H/E, PAS section which showed reddish brown granular casts. They were confirmed with halls histochemical stain that showed emerald green staining in bile casts. It is observed in some studies that Hall’s stain underestimated the number of bile casts and a certain concentration of bilirubin was needed for it to be interpreted as positive (15). In present study also we observed that Hall’s stain showed variable positivity within the same renal biopsy and between different cases, possibly as a result of different concentrations of serum bilirubin as well as different GFR values and urinary stasis.

Various studies have been done on renal pathological changes in hepatic failure either due to acute or chronic causes. Hepatorenal syndrome is thought to be the most important cause of renal dysfunction in hepatic failure. However, studies by van Slambrouk and Nayak et al. have suggested other findings including bile cast nephropathy (1,13). An important difference from the present studies is the striking number of toxin-induced liver damage. Six cases of paraquat and 2 cases of yellow phosphorus poisoning which resulted in bile cast nephropathy have been studied. Paraquat poisoning has variable effects at different doses and its toxicity to the liver is well known. In present study also most of the cases (5/6) showed cholestasis and one case showed submassive necrosis. However, the effect of paraquat in renal biopsy has not been widely reported. Few papers demonstrate acute tubular injury alone as a cause for renal dysfunction (16,17). We have found a large number of such patients to demonstrate bile cast nephropathy. This could prove an important factor in the management of such patients.

In conclusion, current understanding of cholemic nephrosis is incomplete due to the lack of kidney biopsy studies in hepatic failure. This study proves the importance of acute kidney injury along with bile cast nephropathy as a reason for the same with varied hepatotoxic etiologies, especially paraquat and yellow phosphorus. A larger volume of antemortem and post mortem studies are essential to avoid ignorance of this old forgotten entity.

## Conflict of INTEREST

The authors declare no conflict of interest.

## References

[ref-1] Slambrouck Charles M., Salem Fadi, Meehan Shane M., Chang Anthony (2013). Bile cast nephropathy is a common pathologic finding for kidney injury associated with severe liver dysfunction. Kidney Int.

[ref-2] Wadei Hani M., Mai Martin L., Ahsan Nasimul, Gonwa Thomas A. (2006). Hepatorenal syndrome: pathophysiology and management. Clin J Am Soc Nephrol.

[ref-3] Salerno Francesco, Gerbes Alexander, Ginès Pere, Wong Florence, Arroyo Vicente (2007). Diagnosis, prevention and treatment of hepatorenal syndrome in cirrhosis. Gut.

[ref-4] McCormick P. A., Donnelly Carmel (2008). Management of hepatorenal syndrome. Pharmacol Ther.

[ref-5] Nazar André, Pereira Gustavo Henrique, Guevara Mónica, Martín-Llahi Marta, Pepin Marie-Noëlle, Marinelli Marcella, Solá Elsa, Baccaro María Eugenia, Terra Carlos, Arroyo Vicente, Ginès Pere (2010). Predictors of response to therapy with terlipressin and albumin in patients with cirrhosis and type 1 hepatorenal syndrome. Hepatology.

[ref-6] Thompson L L, Frazier W D, Ravdin I S (1940). The renal lesion in obstructive jaundice. Am J Med Sci.

[ref-7] Sequeira Adrian, Gu Xin (2015). Bile cast nephropathy: an often forgotten diagnosis. Hemodial Int.

[ref-8] Pitlick Mitchell, Rastogi Prerna (2017). All That Glitters Yellow Is Not Gold: Presentation and Pathophysiology of Bile Cast Nephropathy. Int J Surg Pathol.

[ref-9] Topuzlu C., Stahl W. M. (1966). Effect of bile infusion on the dog kidney. N Engl J Med.

[ref-10] Gollan J. L., Billing B. H., Huang S. N. (1976). Ultrastructural changes in the isolated rat kidney induced by conjugated bilirubin and bile acids. Br J Exp Pathol.

[ref-11] Fickert Peter, Krones Elisabeth, Pollheimer Marion J., Thueringer Andrea, Moustafa Tarek, Silbert Dagmar, Halilbasic Emina, Yang Min, Jaeschke Hartmut, Stokman Geurt, Wells Rebecca G., Eller Kathrin, Rosenkranz Alexander R., Eggertsen Gosta, Wagner Carsten A., Langner Cord, Denk Helmut, Trauner Michael (2013). Bile acids trigger cholemic nephropathy in common bile-duct-ligated mice. Hepatology.

[ref-12] Krones Elisabeth, Wagner Martin, Eller Kathrin, Rosenkranz Alexander R., Trauner Michael, Fickert Peter (2015). Bile acid-induced cholemic nephropathy. Dig Dis.

[ref-13] Nayak Suman Lata, Kumar Manoj, Bihari Chhagan, Rastogi Archana (2017). Bile Cast Nephropathy in Patients with Acute Kidney Injury Due to Hepatorenal Syndrome: A Postmortem Kidney Biopsy Study. J Clin Transl Hepatol.

[ref-14] Heyman Samuel N., Darmon David, Ackerman Zvi, Rosenberger Christian, Rosen Seymour (2014). Bile cast nephropathy. Kidney Int.

[ref-15] Foshat Michelle, Ruff Heather M., Fischer Wayne G., Beach Robert E., Fowler Mark R., Ju Hyunsu, Aronson Judith F., Afrouzian Marjan (2017). Bile Cast Nephropathy in Cirrhotic Patients: Effects of Chronic Hyperbilirubinemia. Am J Clin Pathol.

[ref-16] Soontornniyomkij V., Bunyaratvej S. (1992). Fatal paraquat poisoning: a light microscopic study in eight autopsy cases. J Med Assoc Thai.

[ref-17] Ravichandran R., Amalnath Deepak, Shaha Kusa K., Srinivas B. H. (2020). Paraquat Poisoning: A Retrospective Study of 55 Patients From a Tertiary Care Center in Southern India. Indian J Crit Care Med.

